# Development of a Simple and Powerful Analytical Method for Formaldehyde Detection and Quantitation in Blood Samples

**DOI:** 10.1155/2020/8810726

**Published:** 2020-12-30

**Authors:** Yong-Hyun Kim, Jeongsik Park

**Affiliations:** ^1^Jeonbuk Department of Inhalation Research, Korea Institute of Toxicology, Jeongeup 56212, Republic of Korea; ^2^Human and Environmental Toxicology, University of Science and Technology, Daejeon 34113, Republic of Korea

## Abstract

Human beings are easily exposed to formaldehyde (FA) in a living environment. Entry of FA into the human body can have adverse effects on human health, depending on the FA concentration. Thus, a quantitative analysis of FA in blood is necessary in order to estimate its effect on the human body. In this study, a simple and rapid analytical method for the quantitation of FA in blood was developed. The total analysis time, including the pretreatment procedure, was less than 20 min. To ensure a stable analysis, blood samples were stabilized using tripotassium ethylenediaminetetraacetic acid solution, and FA was selectively derivatized using 2,4-dinitrophenylhydrazine as pretreatment procedures. The pretreated samples were analyzed using a high-performance liquid chromatography-UV system, which is the most common choice for analyzing small-molecule aldehydes like formaldehyde. Verification of the pretreatment methods (stabilization and derivatization) using FA standards confirmed that the pretreatment methods are highly reliable in the calibration range 0.012–5.761 ng *μ*L^–1^ (slope = 684,898, *R*^2^ = 0.9998, and limit of detection = 0.251 pg·*μ*L^–1^). Analysis of FA in the blood samples of a Yucatan minipig using the new method revealed an average FA concentration of 1.98 ± 0.34 ng *μ*L^–1^ (*n* = 3). Blood samples spiked with FA standards were analyzed, and the FA concentrations were found to be similar to the theoretical concentrations (2.16 ± 0.81% difference). The method reported herein can quantitatively analyze FA in blood at a sub-nanogram level within a short period of time and is validated for application in blood analysis.

## 1. Introduction

Formaldehyde (FA) is a toxin that can cause irritation, immune sensitization, and carcinogenicity when inhaled by the human body [1–6]. High concentrations of FA in blood may have adverse effects on various organs and may even cause blindness [6–8]. FA is oxidized in the blood to produce formic acid, a metabolite that can cause acidosis by the accumulation of formic acid [9, 10]. Due to its toxicity, FA is still being studied [11–13]. In vivo tests on experimental animals are one of the best methods to test toxicity. In order to accurately evaluate the toxic effects of FA in vivo, reliable sampling and analytical methods are required [14, 15]. Furthermore, since FA in the blood is easily excreted in vitro and has a short half-life (1 to 1.5 mins) [16], it is difficult to confirm the concentration of exogenous FA in the blood [17–19], which may be increased by external factors (i.e., inhalation exposure, percutaneous absorption, etc.).

FA is exposed to human inhalation from a variety of environments [20–22]. In 2010, the World Health Organization (WHO) established an indoor air quality guideline for exposure to FA of 0.1 mg/m^3^ (0.08 ppm) for all 30 min periods at lifelong exposure [23]. However, high concentrations of FA are generated from building materials, furniture (made of pressed-wood products), insulation materials, and coating materials, and the occupational exposure to FA is still a problem [24–27]. It is important to prevent the exposure of FA because the cumulative exposure of FA can cause various toxicity as well as genetic damage [1–6, 28, 29]. In order to reduce the concentrations of FA exposed in the living environment, the exposure evaluation of various materials (which can emit FA) should be carried out first. In addition, the FA occupational exposure should be reduced by improving the working environments (conditions) using air purification and ventilation systems [30–34]. Nevertheless, in order to evaluate the adverse effects of FA inhaled into the human body, analytical techniques capable of accurate quantitation for FA in biological samples such as blood are required.

Research on FA in blood has been mainly focused on the toxic effects of FA. Pretreatment via derivatization, followed by chromatography, are commonly used to quantify FA in blood [17, 35, 36]. However, it is difficult to find validation data from previous studies that guarantee the reliability of the analytical method to quantify FA in blood. In addition, expensive instruments and complex analytical procedures were used in the previous studies [17, 18, 35, 36].

In this study, a standardized analytical method for the quantitation of FA in blood was established. The study intends to maximize the reliability and efficiency of bio-based research related to FA. Through simple pretreatment, use of a common analytical instrument, and acquisition of validation data, a reliable analytical method for the quantification of FA in blood was standardized.

## 2. Materials and Methods

### 2.1. Preparation of FA Standards and Blood Samples

#### 2.1.1. Working Standards

The primary standard FA (37% purity) was purchased from Sigma-Aldrich (MO, USA). The working standards were prepared by a gravimetric dilution of the primary standard using distilled water. In case of the first working standard, four different concentrations were prepared by mixing the primary standard with distilled water. The final working standards were prepared by dilution of the first point (the lowest level: 403 ng *μ*L^–1^) of the first working standards with distilled water to generate seven different concentrations of FA, namely, 0.403, 2.02, 4.03, 10.1, 20.2, 40.3, and 202 ng *μ*L^−1^ (Table 1).

#### 2.1.2. Blood Samples

The Yucatan minipig (minipig, 10 weeks, male) obtained from Optipharm (Osong, Republic of Korea) was used in these experiments. Blood samples (2 mL) were collected from the ear vein of the minipig into a sample tube containing ethylenediaminetetraacetic acid (FL Medical, Torreglia, Italy) at thrice the volume of the blood sample. The blood sample in the sample tube was immediately pretreated for stabilization.

#### 2.1.3. Spiked Samples

The first working standards and the blood samples were used to prepare the spiked samples. Briefly, 2 *μ*L of the first working standards (4,033, 10,083, and 20,165 ng *μ*L^–1^) was spiked into a 2 mL electrolytic polishing tube containing 200 *μ*L blood sample. The prepared spiked samples were immediately subjected to pretreatment for FA stabilization.

### 2.2. Instrumentation

**Figure 1:**
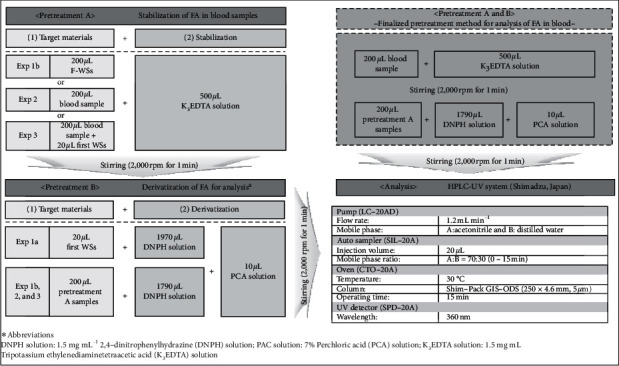
Schematic diagram of the standardized analytical method for the quantitative analysis of FA in blood.

**Figure 2:**
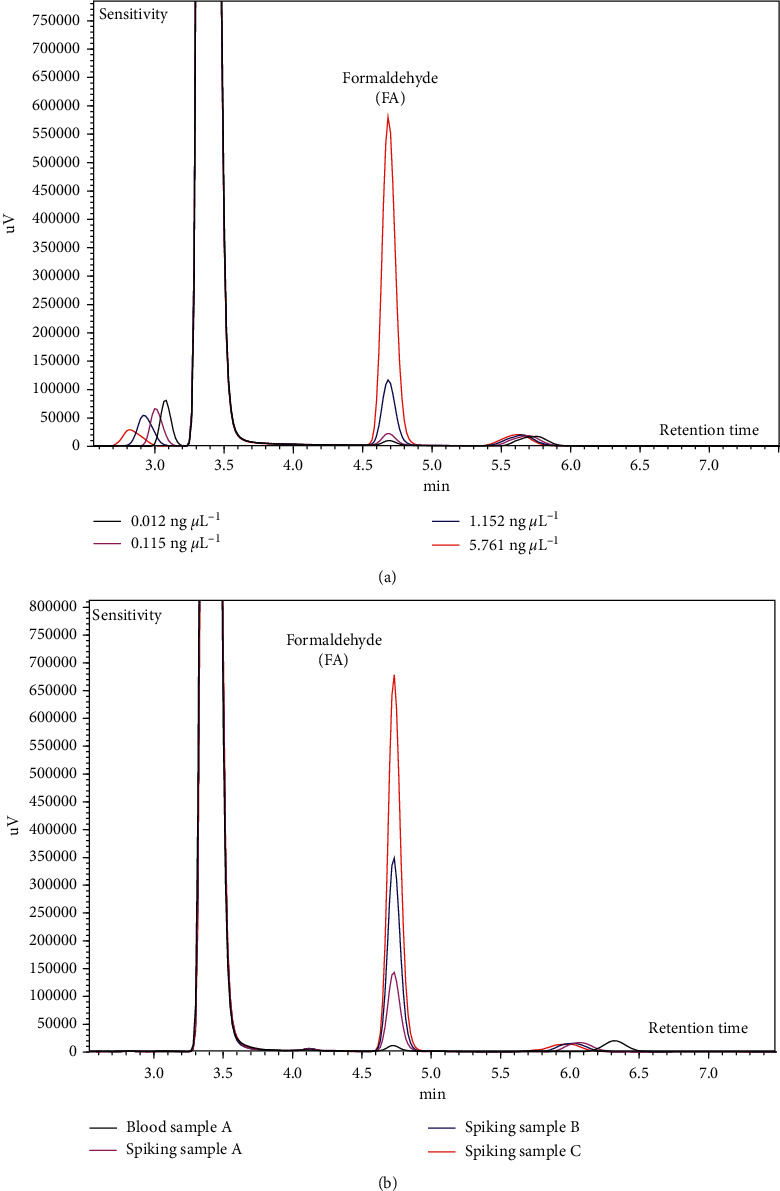
Chromatograms of FA with different experiments. (a) Exp 1b: chromatograms of FA in the final working standards. (b) Exp 2 and 3: chromatograms of FA in blood sample A and spiked samples.

All the samples containing FA were analyzed on an HPLC-UV system (LC-2010, Shimadzu, Japan) equipped with an auto sampler (SIL-20A), pump (LC-20AD), oven (CTO-20A), and UV detector (SPD-20A). The instrumental parameters for minimizing FA interference and achieving optimal separation were obtained. A fixed sample volume of 20 *μ*L was injected into the HPLC system through the auto sampler. FA was separated on a Shim-Pack GIS-ODS column (length: 25 mm, diameter: 4.6 mm, particle size: 5 *μ*L; Shimadzu, Japan) using a mobile phase of acetonitrile–distilled water (7 : 3 (v/v)) at a flow rate of 1.2 mL·min^−1^ at 30°C (maintained by the oven). The total run time was 15 min. The separated FA was detected by the UV detector at a wavelength of 360 nm (Figure 1). The chromatograms obtained from each of all three experiments are presented in Figure 2.

### 2.3. Experimental Scheme

In this study, FA was analyzed in standard solutions and blood samples. First, pretreatment approaches to ensure (a) stabilization of FA in blood samples (pretreatment A) and (b) selectivity of FA detection via derivatization of FA (pretreatment B) were established and validated. Following this, FA in the blood samples was analyzed. In addition, analysis of blood samples spiked with standard FA solution strengthened the validation of the analytical method presented in this study.

  Exp 1a (determination of a reliable calibration range for the analysis of FA in blood): FA in all the samples was derivatized with 2,4-dinitrophenylhydrazine (DNPH) and analyzed on a high-performance liquid chromatography (HPLC)-UV system. FA working standards (20 *μ*L) were injected into a 2 mL vial filled with 1,970 *μ*L DNPH solution (1.5 mg·mL^−1^) and 10 *μ*L perchloric acid (PCA) solution (7%). The prepared FA samples were then stirred immediately at 2,000 rpm for 1 min and analyzed on the HPLC-UV system. Based on the results, a reliable calibration range was assessed.
  Exp 1b (blood pretreatment methods containing FA stabilization and derivatization): FA stabilization was conducted as a pretreatment step to minimize the interference from blood. FA working standards (200 *μ*L) were injected into a 2 mL vial filled with 500 *μ*L tripotassium ethylenediaminetetraacetic acid (K_3_EDTA) solution (1.5 mg mL^–1^) and was stirred at 2,000 rpm for 1 min. Stabilized FA samples (20 *μ*L) were injected into a 2 mL vial filled with 1,970 *μ*L DNPH solution (1.5 mg mL^–1^) and 10 *μ*L perchloric acid (PCA) solution (7%). The prepared FA samples were then stirred immediately at 2,000 rpm for 1 min and analyzed on the HPLC-UV system. Based on the results, the blood pretreatment method for the analysis of FA in blood was evaluated.
  Exp 2 (blood sample analysis): Blood samples from the minipig were pretreated and analyzed as described in Exp 1b. The effectiveness of the pretreatment methods was validated from the analysis of FA in blood samples.
  Exp 3 (spiked sample analysis): Blood samples (three concentrations) of the minipig were spiked with FA standards and analyzed on the HPLC-UV system. The effectiveness of the analytical method was tested through the analysis of the spiked samples.

All these procedures (Exp 1 to 3) for FA analysis are summarized in Table 2 and Figure 1. Sample preparation and instrumental parameters are described in detail in Sections 2.1 and 2.2 and summarized in Table 1.

## 3. Results and Discussion

### 3.1. Analysis of FA Standards (Exp 1)

In Exp 1, reliable FA calibration range of the HPLC-UV system and blood pretreatment methods were assessed and validated using FA working standards (Exp 1a: determination of FA calibration range after DNPH derivatization and Exp 1b: test of the blood pretreatment methods containing FA stabilization).

**Figure 3:**
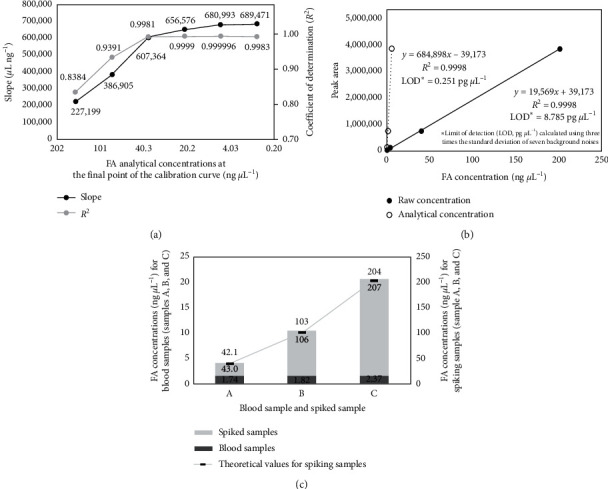
FA analysis in standards and blood samples. (a) Exp 1a: FA calibration in different concentration ranges. (b) Exp 1b: FA calibration and limit of detection. (c) Exp 2 and 3: analysis of spiked and unspiked blood samples.

  Exp 1a: Based on the final analytical concentrations, nine-point calibration data for FA were obtained in the range 0.004–202 ng *μ*L^–1^. The calibration curves were generated by differently setting the highest concentration of the calibration points (4, 5, 6, 7, 8, and 9 points) (Figure 3(a)). At the nine-point calibration curve with the highest concentration of 202 ng *μ*L^–1^, the slope and *R*^2^ of FA were 227,199 *μ*L ng^−1^ and 0.8384, respectively. These values increased with decreasing concentration of the last calibration point. For the calibration curve below 20.2 ng *μ*L^–1^ (4-, 5-, and 6-point calibration curves), FA exhibited high sensitivity (675,680 ± 17,079 *μ*L ng^−1^) and a fairly good linearity (0.9994 ± 0.0010). In addition, the sensitivity, which is used to determine the FA concentration in blood samples, was stable in the range 0.004–20.2 ng *μ*L^–1^. Thus, based on the final analytical concentration, it is important to conduct FA analysis below 20.2 ng *μ*L^–1^ to obtain reliable quantitative data in this analytical system (DNPH derivatization–HPLC-UV system).
  Exp 1b: FA working standards were subjected to pretreatment A for stabilizing the blood samples. Then, FA was derivatized via pretreatment B, and the FA working standards were analyzed on the HPLC-UV system. The slope and *R*^2^ values for the analysis of 0.012 to 761 ng *μ*L^–1^ FA (based on the final analytical concentrations) were 684,898 *μ*L ng^−1^ and 0.9998, respectively. The slope of FA obtained from Exp 1b (after pretreatments A and B) was similar to that of Exp 1a (below 20.2 ng *μ*L^–1^ calibration concentrations) (Figure 3(b)). This indicates that there is no interference on the FA analysis during pretreatment A.

Through Exp 1, it was confirmed that reliable FA quantitation was possible by selectively derivatizing and quantitative analysis of FA in blood samples using only an external quantitative method (external calibration method) without internal standards [37, 38].

### 3.2. Analysis of Blood Samples and Spiked Samples (Exp 2 and 3)

In Exp 2, the minipig blood samples were subjected to pretreatments A and B and were analyzed on the HPLC-UV system. The mean FA concentration in the minipig blood samples was 1.98 ± 0.34 ng *μ*L^–1^ (Figure 3(c)). FA concentrations in blood samples of the human, monkey, and rat are in the range 2–3 ng *μ*L^–1^ [39]. The FA concentration in the minipig blood was similar to that in humans and other animals (i.e., monkey, rat, etc.), and it was confirmed that the difference in the FA concentration in the blood samples of different species was not relatively large.

In Exp 3, the effectiveness and reliability of this simple analytical method for FA quantitation in blood were verified by spiking the samples and then comparing the FA concentrations in them with the theoretical values. The FA concentrations in the spiked samples were 43.0, 106, and 207 ng *μ*L^–1^, which were similar to the theoretical values of 42.1, 103, and 204 ng *μ*L^–1^, with a mean difference of 2.16 ± 0.81% (Figure 3(c)).

(1) $$\begin{array}{c} \text{Percent difference }\left(\%\right)=\;\frac{|\text{FA concentration }\left(\text{measured}\right)\;-\text{ FA concentration }\left(\text{theoretical}\right)|}{\text{FA concentration }\left(\text{theoretical}\right)}\times 100. \end{array}$$

The above results confirm that the pretreatment methods are highly reliable in selectively and accurately analyzing FA in blood.

### 3.3. Finalized Method for FA Analysis in Blood Samples

The analytical method for FA quantitation in blood presented in this study has several critical advantages: (1) this method uses the relatively commonly available low-cost HPLC-UV system, (2) the pretreatment procedures for analyzing FA in blood are simple and require a short time (less than 20 min), and (3) the reliability of the method was maximized by the verification experiments (Exps 1, 2, and 3). The finalized method for FA analysis is presented in Figure 1 and compared with some previous studies (Table 3).

Heck et al. quantified FA in blood using a gas chromatography–mass spectrometry (GC–MS) system [35]. In this study, the blood samples obtained from Fischer-344 were derivatized using pentafluorophenylhydrazine and were analyzed on the GC–MS system after incubation and extraction procedures. However, this method has certain shortcomings. The dilution factor in the FA pretreatment process is not clearly indicated, and the detection limit data cannot be confirmed [35]. In addition, the reliability of the analytical method, such as analytical reproducibility, is poor since there is no pretreatment procedure for stabilizing FA in the blood. Luo et al. analyzed FA in human blood plasma using HPLC equipped with a fluorescence detector [36]. Complex and time-consuming pretreatment procedures such as vortexing, heating, cooling, rinsing, centrifugation, and drying were required for the FA quantitation in the blood plasma samples. However, their analytical method involved no stabilization pretreatment procedure. In addition, the limit of detection (LOD) in the study by Luo et al. was 460 pg·*μ*L^–1^, which was ∼1000 times higher than that in the current study [36]. Kleinnijenguis et al. obtained blood samples from a SD rat, and the stabilization and derivatization of FA in the blood samples were performed in a manner similar to this study [17]. However, the pretreatment time in their study was ∼9 times longer than that in our study. The expected recovery (associated with the dilution factor in pretreatment procedures) is ∼3 times lower, and the detection limit is ∼6 times higher [17]. Moreover, the analytical method established by Kleinnijenguis et al. used the latest expensive analytical instruments such as tandem MS (MS/MS) [17], while our method utilizes the commonly available HPLC-UV system to analyze sub-ppb levels of FA in blood with high reliability.

## 4. Conclusion

Although there are many studies reporting the adverse health effects of FA, there is a lack of study on the FA analysis in blood. Analysis of FA is important for understanding the behavior of FA in the human body. In this study, a simple analytical method for the quantitation of FA in blood was developed and standardized through method validation experiments. FA in blood was stabilized using K_3_EDTA, and the FA detection selectivity was enhanced by FA derivatization using 2,4-DNPH. When the FA working standards were pretreated and analyzed using the developed method, reliable calibration data were obtained with *R*^2^ values greater than 0.999. Moreover, the detection limit of FA was relatively low (0.251 pg·*μ*L^−1^), which corresponds to a fairly good analytical sensitivity. The blood samples of the minipig were analyzed, and the mean FA concentration in the blood samples was 1.98 ± 0.34 ng *μ*L^–1^. Analysis of the spiked samples (known concentrations of FA working standards spiked into minipig blood samples) confirmed that the FA concentrations in the spiked samples were similar to the theoretical concentrations, with a difference of 2.16 ± 0.81%. Thus, the analytical method presented in this study could minimize the interference from blood components and could be used to detect sub-ppb levels of FA in blood. Moreover, the analytical method involves simple pretreatment procedures and uses one of the most common analytical instruments (HPLC-UV system). The analytical method for FA quantitation in blood samples was standardized. We believe that our study will contribute to the studies related to the health effects of FA.

## Figures and Tables

**Table 1:** Preparation of FA standards and blood samples.

Order	Mixing volume (*μ*L)		Concentration (ng *μ*L^−1^)
	Primary standard^a^	Distilled water	(ng *μ*L^−1^)
A. First working standards (first WSs)			
1	2	1,998	403
2	10	1,990	2,017
3	20	1,980	4,033
4	50	1,950	10,083
5	100	1,900	20,165

	First point of first WSs	Distilled water	(ng *μ*L^−1^)
B. Final working standards (F-WSs)			
1	2	1,998	0.403
2	10	1,990	2.02
3	20	1,980	4.03
4	50	1,950	10.1
5	100	1,900	20.2
6	200	1,800	40.3
7	1,000	1,000	202

C. Blood samples
Experimental animal	Yucatan minipig		
Sex	Male		
Age	10 weeks		
Number of animals	Three		
Blood sampling point	Ear vein		
Blood sampling method	Intravenous (IV)		
Sampling volume	Each 2 mL		
Blood sample codes	Blood samples A, B, and C		

^a^The purchased primary standard of formaldehyde was 37% pure (403,300 ng *μ*L^−1^).

**Table 2:** Experimental scheme for the development and validation of the pretreatment method for FA analysis in blood samples.

Experimental codes	Contents	Pretreatment method	Target material^a^	Concentration (ng *μ*L^−1^)		Number of calibration points	Dilution factor in pretreatment procedures
				Standards^b^	Analytical^c^		
Exp 1a	Analysis of FA in standards	Pretreatment B	20 *μ*L first WSs and F-WSs	0.40 to 20,165	0.004 to 202	9 points	100
Exp 1b			200 *μ*L F-WSs	0.40 to 202	0.012 to 5.761	4 points	35
Exp 2	Blood sample analysis	Pretreatments A and B	200 *μ*L blood sample	NA	NA	NA	
Exp 3	Spiking sample analysis (blood sample + standards)		200 *μ*L blood sample + 2 *μ*L first WSs	4,033 to 20,165 for first WSs	1.22 to 6.09 for first WSs	3 points	-

^a^WS: working standard; F-WS: final working standard. ^b^Exp 1a: 0.40, 2.02, 10.08, 20.17, 403, 2,017, 4,033, 10,083, and 20,165 ng *μ*L^–1^; Exp 1b: 0.40, 4.03, 40.33, and 201.65 ng *μ*L^–1^; Exp 3 : 4,033, 10,083, and 20,165 ng *μ*L^–1^. ^c^Exp 1a: 0.004, 0.020, 0.1008, 0.2017, 4.03, 20.17, 40.33, 100.83, and 201.65 ng *μ*L^–1^; Exp 1b: 0.012, 0.115, 1.152, and 5.761 ng *μ*L^–1^; Exp 3 : 1.22, 3.05, and 6.09 ng *μ*L^–1^. NA: not available.

**Table 3:** Overview of the parameters for the analysis of FA in blood.

Order	Blood sample	Analytical system		Detection limits	Pretreatment				Reference
	Source	Separation	Detection	(pg *μ*L^–1^)	Stabilization	Derivatizer	Others	Dilution factor	
1	Yucatan minipig	HPLC	UV	0.251 (LOD)	○ (using K_3_EDTA)	DNPH	– Vortexing (2 min)	35	This study
2	SD rat	HPLC	MS/MS	1.5	○ (using K_3_EDTA)	DNPH	– Vortexing (above 3 min) – Room temp. Reaction (15 min)	91	Kleinnijenguis et al. [17]
3	Human (blood plasma)	HPLC	FL	460 (LOD)	×	Ampicillin	– Vortexing (1.5 min) – Heating (1 ) – Cooling – Rinsing – Centrifugation (5 min) – Drying	Above 24.3	Luo et al. [36]
4	Fischer-344 rat	GC	MS	Not available	×	PFPH	– Incubation (2 h) – Extraction	Above 9.71	Heck et al. [35]

DNPH: 2,4-dinitrophenylhydrazine; FL: fluorescence; GC: gas chromatography; HPLC: high-performance liquid chromatography; LOD: limit of detection; MS: mass spectrometry; PFPH: pentafluorophenylhydrazine; SD rat: Spragure–Dawley rat.

## Data Availability

The data used to support the findings of this study are available from the corresponding author upon request.
